# SMetABF: A rapid algorithm for Bayesian GWAS meta-analysis with a large number of studies included

**DOI:** 10.1371/journal.pcbi.1009948

**Published:** 2022-03-14

**Authors:** Jianle Sun, Ruiqi Lyu, Luojia Deng, Qianwen Li, Yang Zhao, Yue Zhang

**Affiliations:** 1 Department of Bioinformatics and Biostatistics, School of Life Sciences and Biotechnology, Shanghai Jiao Tong University, Shanghai, China; 2 Department of Biostatistics, Nanjing Medical University School of Public Health, Nanjing, Jiangsu, China; Heidelberg University, GERMANY

## Abstract

Bayesian methods are widely used in the GWAS meta-analysis. But the considerable consumption in both computing time and memory space poses great challenges for large-scale meta-analyses. In this research, we propose an algorithm named SMetABF to rapidly obtain the optimal ABF in the GWAS meta-analysis, where shotgun stochastic search (SSS) is introduced to improve the Bayesian GWAS meta-analysis framework, MetABF. Simulation studies confirm that SMetABF performs well in both speed and accuracy, compared to exhaustive methods and MCMC. SMetABF is applied to real GWAS datasets to find several essential loci related to Parkinson’s disease (PD) and the results support the underlying relationship between PD and other autoimmune disorders. Developed as an R package and a web tool, SMetABF will become a useful tool to integrate different studies and identify more variants associated with complex traits.

## Author summary

MetABF is a Bayesian GWAS meta-analysis framework but the efficiency is restricted by the number of studies included. In this article, we propose SMetABF by introducing SSS, an improved edition of traditional MCMC, to speed the MetABF algorithm. We develop an R package and a web tool based on R Shiny to make SMetABF practical for biomedical research. Comparing with the exhaustive approach and MCMC, we validate the effectiveness of SSS in terms of speed and accuracy through simulations. We applied SMetABF to identify several important variants associated with Parkinson’s disease and other autoimmune diseases, and explore the relationship between them. We hope this method can benefit future GWAS meta-analyses, help to identify more risk variants associated with complex traits, and improve the prediction of diseases.

## 1 Introduction

Genome-wide association study (GWAS), a powerful tool to find out the associations between genetic variations and phenotypes, has received more and more attention in the field of statistical genetics and epidemiology [1]. Numerous variants, typically many common single nucleotide polymorphisms (SNPs), are identified linked with complex traits. However, since single variant’s genetic effect on polygenic traits is relatively small, large sample sizes are often required to increase the statistical power [2]. Besides, due to the population stratification and other unobserved confounders, the estimated effect sizes in different studies are divided or even contradictory [3]. Therefore, it has become an increasingly essential challenge to make sufficient use of summary statistics derived from a wide range of studies and to attain pooled statistics through meta-analysis [4], especially when the requirement of data security and privacy makes individual-level data increasingly difficult to obtain [5, 6].

Either the fixed-effect model (FEM) [7] or the random-effect model (REM) [8] is conventionally used to derive a pooled effect size, depending on the assumption on heterogeneity [9]. However, the *p*-value is dependent on the sample size and minor allele frequency (MAF) of the variant. Therefore, it is improper to use a single threshold [10]. Besides, the relationships between true effect sizes in different studies are hard to be considered in both FEM and REM [11]. On the contrary, it is easy to involve them into the model as a prior in the Bayesian framework. The Bayesian method is also prevalent for researchers for it is more intuitively explainable [12]. Recently, a promising method based on the Bayesian framework named MetABF has already been proposed [13]. With GWAS summary statistics, it could conveniently estimate the pooled associations between multiple traits and genetic variations in different associated models across studies. However, with the rapidly increasing data of GWAS, the method also confronts the challenge of exponential explosion in both time and space consumption. Since it requires traversing 2*^n^* subsets represented by *n*-dimensional vectors to compute the optimal ABF, the considerable time and memory consumption required makes the computation almost impossible as the number of studies *n* increases.

In this article, we propose SMetABF, a method based on the Markov chain Monte Carlo (MCMC) method and its extension named shotgun stochastic search (SSS) [14] to speed the process of subset selection. SSS is proved to be superior in speed, accuracy, and stability through simulation. Based on SSS, we introduce SMetABF to obtain the maximum ABF in a large-scale meta-analysis quickly. SMetABF is implemented as an R package and the code is available at https://github.com/sjl-sjtu/GWAS_meta.

## 2 Method

### 2.1 Asymptotic Bayes factor

Different from the traditional statistical framework based on the *p*-value for statistical inference, the Bayes factor (BF) is used in Bayesian statistical framework. BF is defined as the relative size of the likelihood to observe data under the null hypothesis (*H*_0_) or the alternative one (*H*_1_),

$$\text{BF}=\frac{P(D|H_{1})}{P(D|H_{0})}=\frac{\int_{\beta }\int_{\gamma }P\left(Y|\beta ,\gamma \right)\pi \left(\beta ,\gamma \right)d\beta d\gamma }{\int_{\gamma }P\left(Y|\beta =0,\gamma \right)\pi \left(\beta =0,\gamma \right)d\gamma },$$

where *D* stands for the data observed, *β* is the effect parameter we are interested in, ***γ*** is the parameter vector of confounders, and *π*(⋅) stands for the prior of *β* and *γ*. In general, BF > 1 means more inclined to accept *H*_1_, and on the contrary, 0 < BF < 1 means more inclined to accept *H*_0_. Since BF is difficult to calculate directly in many studies, an asymptotic Bayes factor (ABF) is proposed as an alternative [10]. If *P*(*Y*|*β*, ***γ***) is replaced by the asymptotic distribution $P(\hat{\beta },\hat{\gamma }|\beta ,\gamma )$, and the marginal prior for *β* instead of the joint prior *π*(*β*, ***γ***) is considered, the probability of obtaining the parameter *β* under a certain hypothesis could replace the probability of observing data *Y*, written as

$$\text{ABF}=\frac{P(\beta |H_{1})}{P(\beta |H_{0})}.$$

For a study aimed to measure the association between several risk factors and specific outcomes, like GWAS, let $\hat{\beta }$ be the estimated size of the association. It is assumed to obey the normal distribution

$$\hat{\beta }∼N(\beta ,\;SE_{\hat{\beta }}^{2}),$$

where *β* is the true effect size of the variant, and $SE_{\hat{\beta }}$ represents the estimated standard error. The true effect size *β* is also assumed to follow a normal distribution

$$\beta ∼N(0,\;\sigma^{2}),$$

where *σ*^2^ represents the prior variance of the true effect size. When *σ* = 0, the distribution of *β* degenerates to a point, which means *β* = 0. In other words, the genetic variant has no effect on the outcome. Under *H*_0_: *σ* = 0, ABF can be calculated as

$$\text{ABF}=\frac{f(\hat{\beta };0,SE_{\hat{\beta }}^{2}+\sigma^{2})}{f(\hat{\beta };0,SE_{\hat{\beta }}^{2})}.$$

where *f*(*x*; *m*, *s*^2^) is the probability density of normal distribution *N*(*m*, *s*^2^) at *x*.

Each study included in the meta-analysis provides an estimated effect size, $\hat{\beta }$. When there are *n* studies in the meta-analysis, let $\hat{\beta }$ be the estimated effect vector

$$\hat{\beta }={\left(\hat{\beta_{1}},\hat{\beta_{2}},\ldots ,\hat{\beta_{n}}\right)}^{T},$$

and $\hat{\beta }$ follows a multivariate normal distribution $N(\beta ,\;V_{\hat{\beta }})$, where *β* stands for the true effect vector, and $V_{\hat{\beta }}$ represents the covariance matrix of the estimated standard errors, expressed as

$$V_{\hat{\beta }}=(\begin{array}{cccc} SE_{1}^{2} & r_{1,2}SE_{1}SE_{2} & \cdots & r_{1,n}SE_{1}SE_{n}\\ r_{1,2}SE_{1}SE_{2} & SE_{2}^{2} & \cdots & r_{2,n}SE_{2}SE_{n}\\ \vdots & \vdots & \vdots & \vdots \\ r_{1,n}SE_{1}SE_{n} & r_{2,n}SE_{2}SE_{n} & \cdots & SE_{n}^{2} \end{array}),$$

in which *SE*_*i*_ is the standard error of $\hat{\beta_{i}}$ in the *i*-th study, and *r*_*i*,*j*_ is the correlation between the *i*-th and *j*-th studies. For each study, the prior effect size is *σ*_*i*_, and the prior correlation coefficient between two studies is *ρ*_*i*,*j*_, then the prior matrix **Σ** is

$$\Sigma =(\begin{array}{cccc} \sigma_{1}^{2} & \rho_{1,2}\sigma_{1}\sigma_{2} & \cdots & \rho_{1,n}\sigma_{1}\sigma_{n}\\ \rho_{1,2}\sigma_{1}\sigma_{2} & \sigma_{2}^{2} & \cdots & \rho_{1,n}\sigma_{2}\sigma_{n}\\ \vdots & \vdots & \cdots & \vdots \\ \rho_{1,n}\sigma_{1}\sigma_{n} & \rho_{1,n}\sigma_{2}\sigma_{n} & \cdots & \sigma_{n}^{2} \end{array}).$$

With the estimated effect vector and covariance matrix of the estimated standard errors, ABF for meta-analysis could be calculated as

$$\text{ABF}=\frac{f(\hat{\beta };0,V_{\hat{\beta }}+\Sigma )}{f(\hat{\beta };0,V_{\hat{\beta }})}.$$

Similarly, *H*_0_ is defined that **Σ** equals to the zero matrix.

### 2.2 Prior

The assumption on the heterogeneity among studies is critical in prior selection. Table 1 provides different models for prior under the assumption that *σ* and *ρ* remain the same for all studies included in the meta-analysis.

**Table 1:** Different models of prior across studies [13].

parameter	name	set
*σ*	null model	*σ*^2^ = 0
	complete model	*σ*^2^ > 0
	subset model	*σ*^2^ > 0 for subset *I* ⊂ {1, …, *n*}
*ρ*	fixed effect	*ρ* = 1
	independent effect	*ρ* = 0
	correlated effect	0 < *ρ* < 1

Since both the null model and the complete model can be regarded as special cases of the subset model, the subset model is adopted in the meta-analysis. But it also brings a tricky issue to determine the optimal subsets. It is preferred to get a higher ABF score in the meta-analysis, for it means that *H*_0_ is harder to be accepted and the probability of the type II error decreases. In other words, it increases the statistical power, which equals one minus the probability of the type II error.

### 2.3 Model selection

#### 2.3.1 Subset-exhaustive

The former study [13] performs model selection by traversing subsets to select the highest ABF, named the subset-exhaustive method (EXH). For a meta-analysis including *n* studies, there are in total 2^*n*^ different subsets. It requires taking all these subsets as a prior one by one to calculate ABF, and then find the optimal one. The time consumed will explode exponentially as the number of studies included increases. At the same time, the memory required to store all subsets (2^*n*^ *n*-dimensional 0-1 vectors) has also expanded dramatically. Therefore, it becomes quite essential to introduce a method to get higher ABF quickly.

#### 2.3.2 MCMC

A commonly used method to quickly find the best subset is the Markov chain Monte Carlo method (MCMC). Here MC^3^ algorithm [15, 16] is used to define the transition function and the Metropolis-Hastings algorithm is used for sampling. The whole process is carried out as Algorithm 1.

**Algorithm 1** MCMC Pseudocode

**Require**: Ω: Universe of subsets.

**Ensure**: *x*: Sample with stable distribution.

 *x*_0_ ← random sample from Ω

 **for** *t* = 0…*T* **do**

  Update *nbd*(*x*_*t*_)                   ⊳ Neighborhood

  $\Pi \left(x_{t}\right)\leftarrow \left\{P_{i\in nbd(x_{t})}=\frac{1}{\left||nbd(x_{t})|\right|},P_{i\notin nbd(x_{t})}=0\right\}$   ⊳ Transition Probability

Generate *y* based on Π(*x*_*t*_)                   ⊳ Alternative Model

  $h=\text{min}\{1,\frac{ABF(y)}{ABF(x_{t-1})}\}$              ⊳ Discriminant Function

Generate *u* from uniform distribution *U*(0, 1)

  **if** *u* < *h* **then**

   *x*_*t*+1_ = *y*

  **else**

   *x*_*t*+1_ = *x*_*t*_

  **end if**

 **end for**

1. Randomly select a subset as the initial prior model *x*_0_, and calculate the ABF.
2. For the current model *x*_*t*_, define the neighborhood as a set constituted of subsets formed by adding or deleting an element from the current subset, as well as the current model itself. The proposal distribution is defined by equalizing the sampling probability of all models in the neighborhood. In other words, the sampling probability of all models in the neighborhood remains equal, and the transition probability of all models outside the neighborhood is 0. Since each model has the same size of the neighborhood, the proposal distribution is symmetric.
3. Generate the alternative prior model *y* according to the transition probability, and then calculate the ABF. The discriminant function is defined as $h=\text{min}\{1,\frac{ABF(y)}{ABF(x_{t-1})}\}$, where *ABF*(*x*) represents the ABF value with subset *x* as the prior.
4. Generate a random number *u* that follows the uniform distribution *U*(0, 1). If *u* < *h*, accept *y* as a new step of *x*_*t*+1_, and otherwise, *x*_*t*+1_ = *x*_*t*_.
5. Repeat steps 2-4 until the maximum number of iterations or stable distribution is reached.

The first half of the entire iterative sequence is used for the warm-up and the second for the final sampling.

#### 2.3.3 Shotgun stochastic search

Here we introduce an extension of MCMC for variable selection named shotgun stochastic search (SSS) [14]. It can be used to fast detect the optimal ABF following the procedures as below (see Algorithm 2):

1. Let Γ donate a set containing up to *B* optimal models. Randomly select the initial model *x*_0_, set Γ = {*x*_0_}, and calculate the score of the model *S*(*x*) = *ABF*(*x*).
2. For the current model *x*_*t*_, define models that add or delete or replace an element from the current subset to constitute the sets Γ^+^, Γ^−^, and Γ^∘^, respectively, and then define the neighborhood
  $$nbd(x_{t})=\Gamma^{+}\cup \Gamma^{-}\cup \Gamma^{\circ }.$$
  Then update Γ = Γ ∪ *nbd*(*x*_*t*_). If |Γ| > *B*, remove (|Γ| − *B*) models with the lowest scores.
3. Sample *x*^+^, *x*^−^, and *x*^∘^ from Γ^+^, Γ^−^, and Γ^∘^, with the score *S*(*x*) as sampling weight, respectively.
4. Then take a sample from *x*^+^, *x*^−^, and *x*^∘^, with the score *S*(*x*) as sampling weight, and let the sample be the new model *x*_*t*+1_.
5. Repeat steps 2-4 until the maximum number of iterations is reached.

**Algorithm 2** SSS Pseudocode

**Require**: Ω: Universe of subsets.

**Ensure**: *x*: Sample with stable distribution.

 *x*_0_ ← random sample from Ω

 Γ = {*x*_0_}

 *S*(*x*)←*ABF*(*x*)

 **for** *t* = 0…*T* **do**

  Constitute Γ^+^, Γ^−^, Γ^∘^

  *nbd*(*x*_*t*_) = Γ^+^ ∪ Γ^−^ ∪ Γ^∘^

  Update Γ = Γ ∪ *nbd*(*x*_*t*_)

  **if** |Γ| > *B* **then**

   Remove (|Γ| − *B*) models with lowest *S*.

  **end if**

  Sample *x*^+^ from Γ^+^, weight = *S*(*x*)

  Sample *x*^−^ from Γ^−^, weight = *S*(*x*)

  Sample *x*^∘^ from Γ^∘^, weight = *S*(*x*)

  Sample *x*_*t*+1_ from *x*^+^, *x*^−^, *x*^∘^, weight = *S*(*x*)

  **if** *x*_*t*+1_ satisfies stable distribution **then**

   break

  **end if**

 **end for**

The former study [13] has provided R code for EXH. Here R functions for meta-analysis by MCMC and SSS are constructed.

## 3 Simulation

### 3.1 The construction of simulated datasets

Several parameters are given to build the simulated datasets, including the incidence of the disease in the population (*p*), the frequency of the major allele of the studied variant (*f*, which equals to 1-MAF under the assumption that there are only two alleles in the SNP), the effect size (odds ratio, OR), and the sample size of both case and control groups (which is assumed to be the same, *n*). For example, suppose A is the risk allele while G is the non-risk allele. If the dominant model is applied, both AA and AG can be considered as equivalent risk genotypes while GG is non-risk. Suppose baseline effect is *α*, the increased effect on prevalence by risk genotype is *θ*, then

P(D|GG)=α,

P(D|AA+AG)=α(1+θ),

where *D* represents the outcome (disease). Then we can get

$$\text{OR}=\frac{P(D|GG)}{P(\bar{D}|GG)}\cdot \frac{P(\bar{D}|AA+AG)}{P(D|AA+AG)}=\frac{1-\alpha }{\alpha }\cdot \frac{\alpha (1+\theta )}{1-\alpha (1+\theta )},$$

and

$$p=f^{2}\alpha +\left(1-f^{2}\right)\alpha \left(1+\theta \right).$$

Then *α* and *θ* can be calculated. According to the Bayes Theorem, the probability of risk and non-risk genotypes in the case and control groups can be calculated.

$$P(GG|D)=\frac{P(D|GG)P(GG)}{P(D)}=\frac{f^{2}}{p},$$

$$P(GG|\bar{D})=\frac{(1-\alpha )f^{2}}{1-p}.$$

And then, the simulated genotypes in both the case and control groups could be randomly generated under binomial distribution. The estimated $\hat{\text{OR}}$ can be calculated. The effect size is defined as *β* = ln OR, and similarly, $\hat{\beta }=\text{ln}\hat{\text{OR}}$. The standard error can be estimated from the contingency table, as $SE_{\hat{\beta }}=\sqrt{\frac{1}{n_{11}}\cdot \frac{1}{n_{12}}\cdot \frac{1}{n_{21}}\cdot \frac{1}{n_{22}}}$.

Suppose there are *N* studies included in the meta-analysis. For each study, the true effect OR_*i*_ obeys the normal distribution *N*(OR, *SE*^2^). The sample size *n*_*i*_ in each study is generated as a random integer in a given range, and *p* and *f* remain the same for all studies. Through the process above, $\hat{\beta_{i}}$ and $SE_{\hat{\beta_{i}}}$ of each study can be estimated.

### 3.2 Results

**Fig 1:**
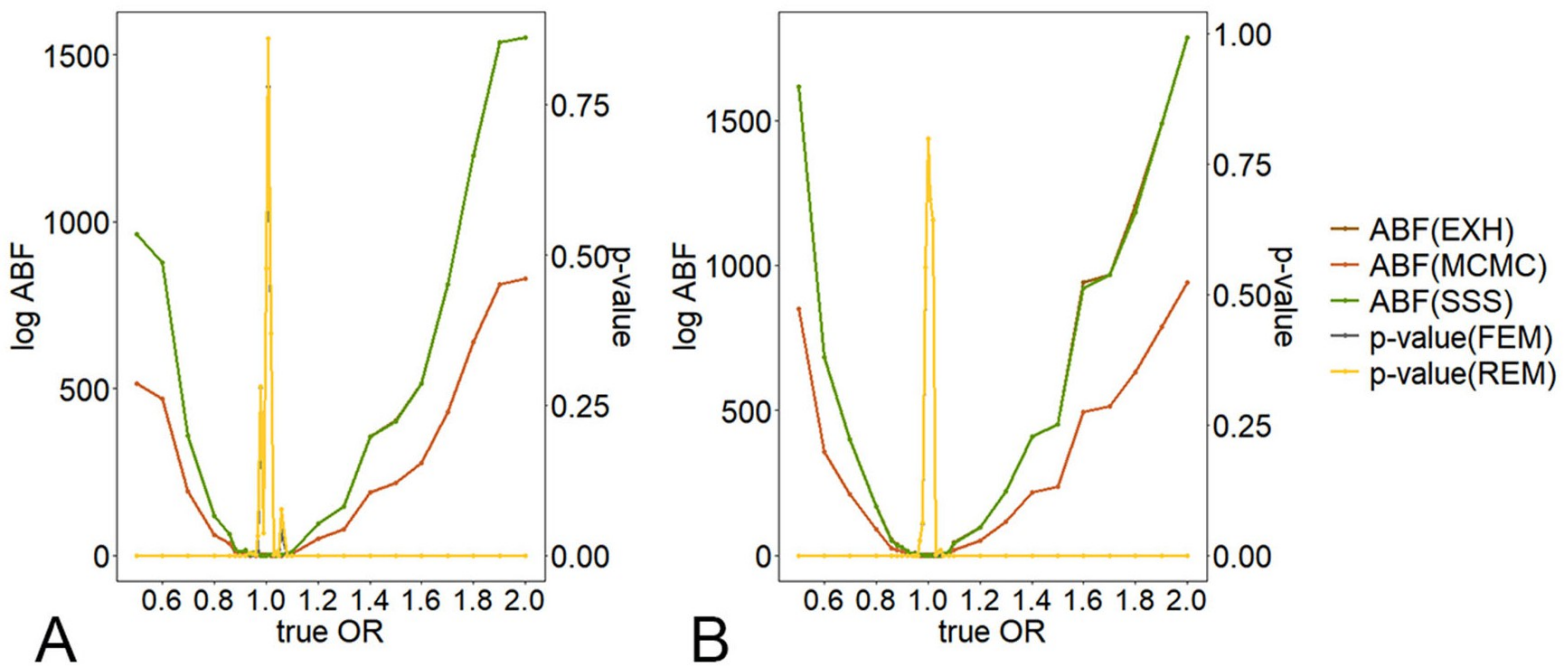
The comparison under various true ORs. Curves representing ABF (EXH) and ABF (SSS) are nearly coincide. Curves representing *p*-value (FEM) and *p*-value (REM) are nearly coincide as well. The parameters are set as follows: *p* = 0.05, *f* = 0.8 (which equally means MAF = 0.2), the number of studies included (*N*) is set to be 20 (Fig 1A) and 25 (Fig 1B) respectively. For the *i*-th study, OR_*i*_ ∼ *N*(OR, 0.01), the sample size *n*_*i*_ is sampled from 100 to 2000 and 100 to 5000, respectively.

The ABFs calculated under different true ORs are shown in Fig 1. The overall trends of the ABF obtained by EXH, MCMC, and SSS remain consistent, corresponding to the *p*-value obtained by the traditional method. When the true OR approaches 1, the *p*-value increases, while the ABF value decreases to 0. However, when the sample size of the study included is small (Fig 1A), the change of *p*-value will be unstable if true OR is near to 1, which will affect the analysis. Besides, the ABF calculated by SSS almost coincides with the optimal ABF curve obtained by EXH, which shows the validity of SMetABF.

**Fig 2:**
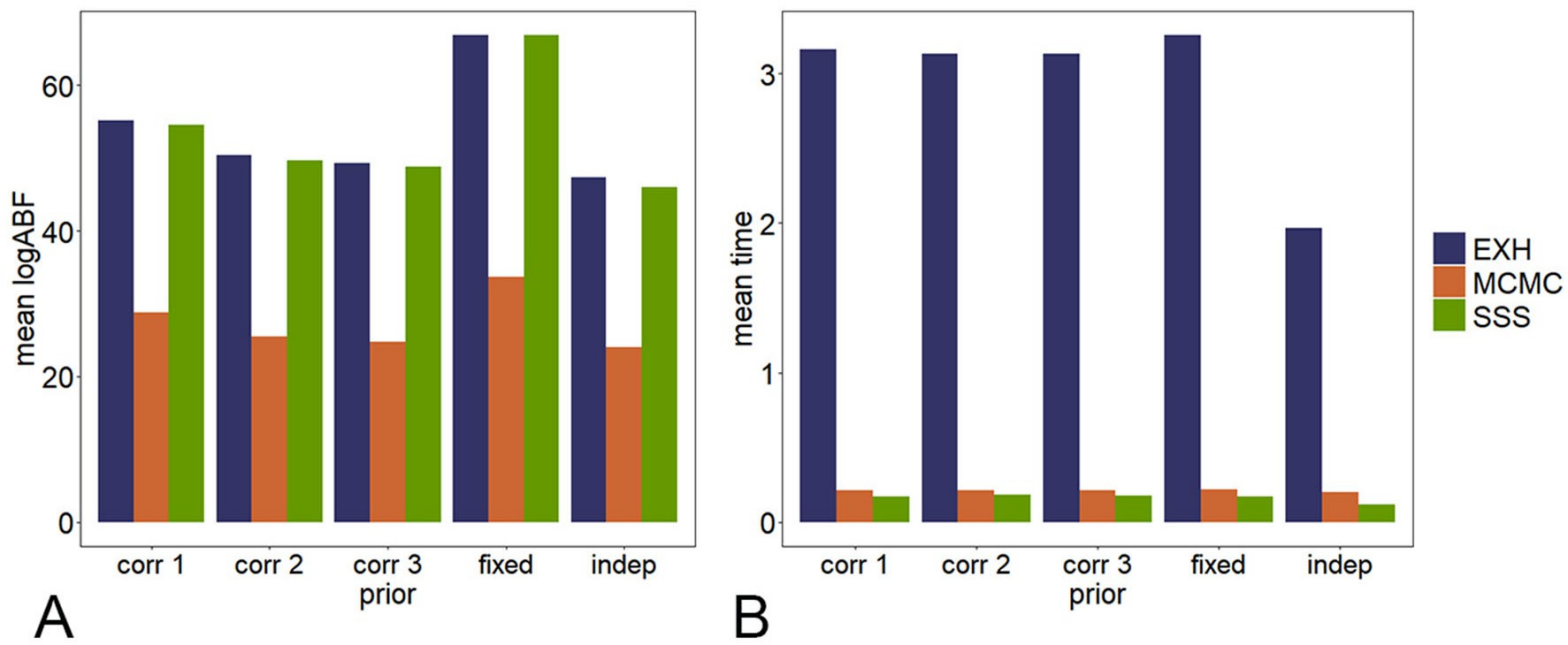
The comparison in accuracy and speed of the three algorithms under different priors. Priors setting: corr 1 (correlated model, *σ* = 0.5, *ρ* = 0.7); corr 2 (correlated model, *σ* = 0.5, *ρ* = 0.3); corr 3 (correlated model, *σ* = 0.8, *ρ* = 0.7); fixed (fixed model, *σ* = 0.5); indep (independent model, *σ* = 0.5).

**Fig 3:**
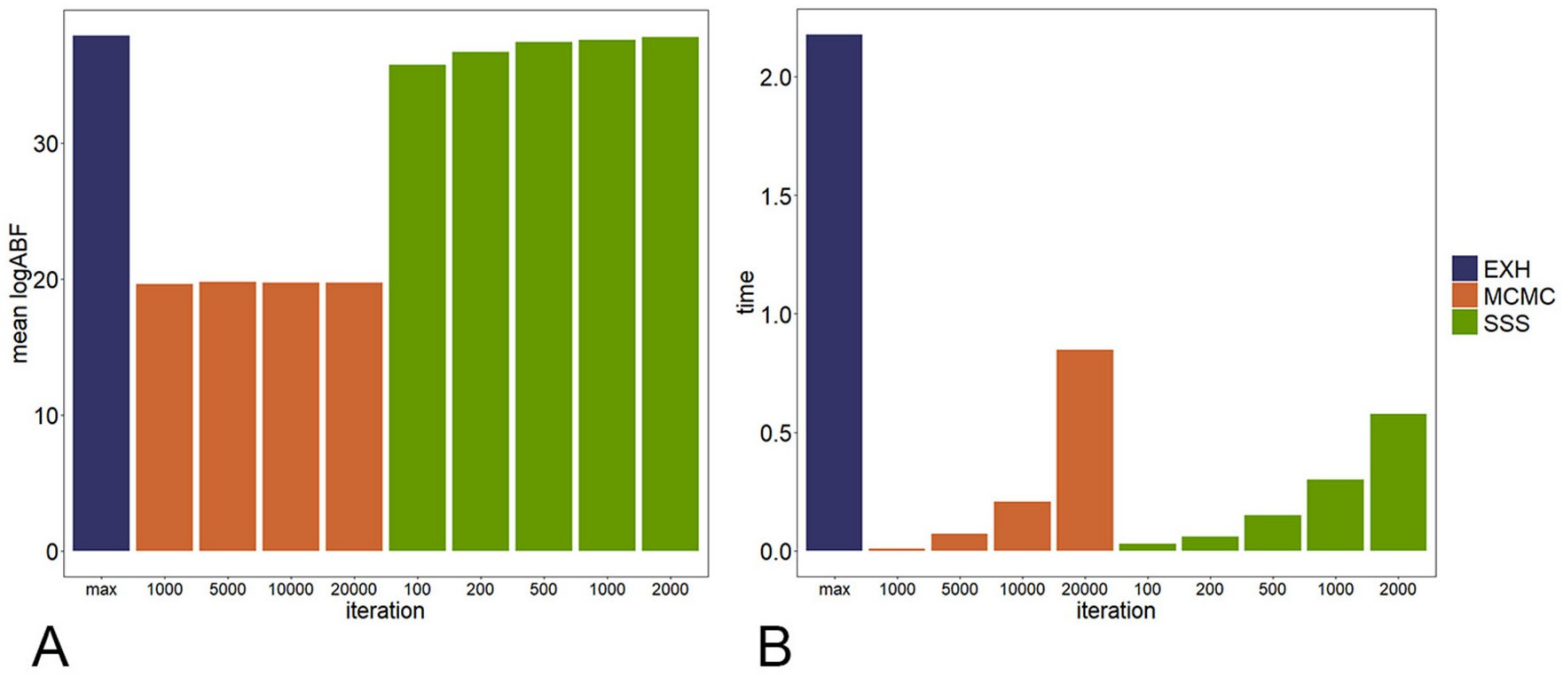
The comparison in accuracy and speed of the three algorithms under different iterations.

Figs 2 and 3 show the performance of each algorithm in accuracy and speed under different priors and iterations, respectively. Since in SSS, ABF is calculated under all models in the neighborhood in one iteration, much more models will be calculated by the SSS with the same number of iterations. Therefore, the number of iterations of SSS is set to be 100, 200, 500, 1000 and 2000; while that of MCMC is set to be 1000, 5000, 10000 and 20000. To compare the averaged ABF and time consumed, the algorithm is repeated 100 times under each condition. The SSS algorithm can reach the maximum ABF in a short time with a small number of iterations. On the contrary, the MCMC algorithm can hardly find the maximum ABF in even longer time.

**Fig 4:**
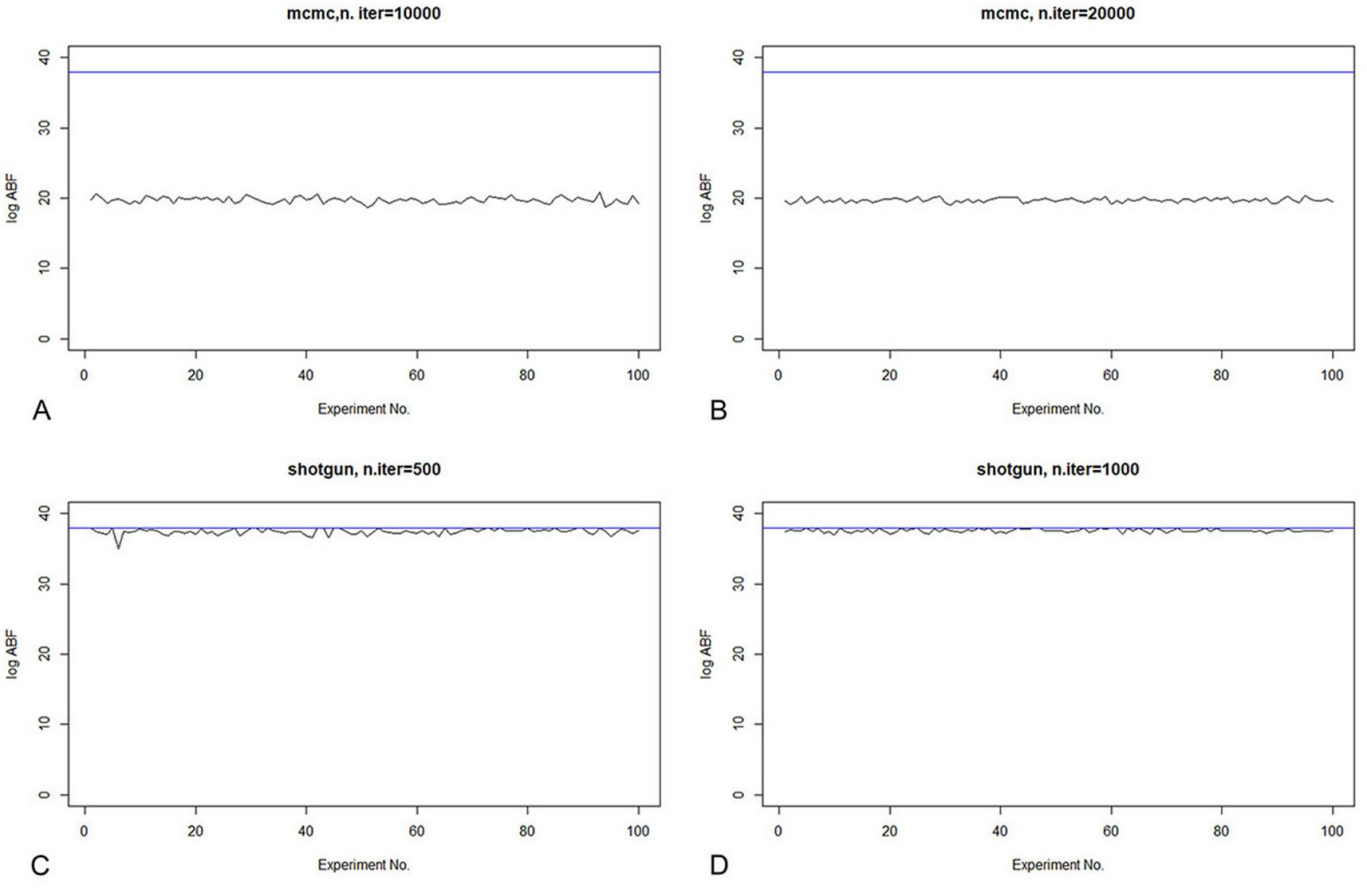
The comparison in stability of MCMC and SSS.

When repeating 100 times of MCMC (10000 and 20000 iterations) and SSS (500 and 1000 iterations), as shown in Fig 4, the ABF values obtained by MCMC are relatively small, while the results of SSS are relatively stable, very close to the maximum ABF.

## 4 Application

### 4.1 Meta-analysis on the variants related to PD and other autoimmune disorders

Here an application is performed to measure the risk variants associated with Parkinson’s disease (PD), a common chronic neurodegenerative disease among the elderly population. Its common clinical manifestations include tremors, slow movement, and disorders in balance and movement posture. PD has been reported to be associated with both genetic variations [17] and environmental factors like personal lifestyles such as smoking and drinking [18, 19], but the detailed mechanism remains unclear. Recent studies discuss the potential relationship between PD and autoimmune disorders [20]. To explore the underlying relationships, we conduct a GWAS meta-analysis across PD and three common autoimmune disorders: inflammatory bowel disease, multiple sclerosis, and systemic sclerosis.

**Fig 5:**
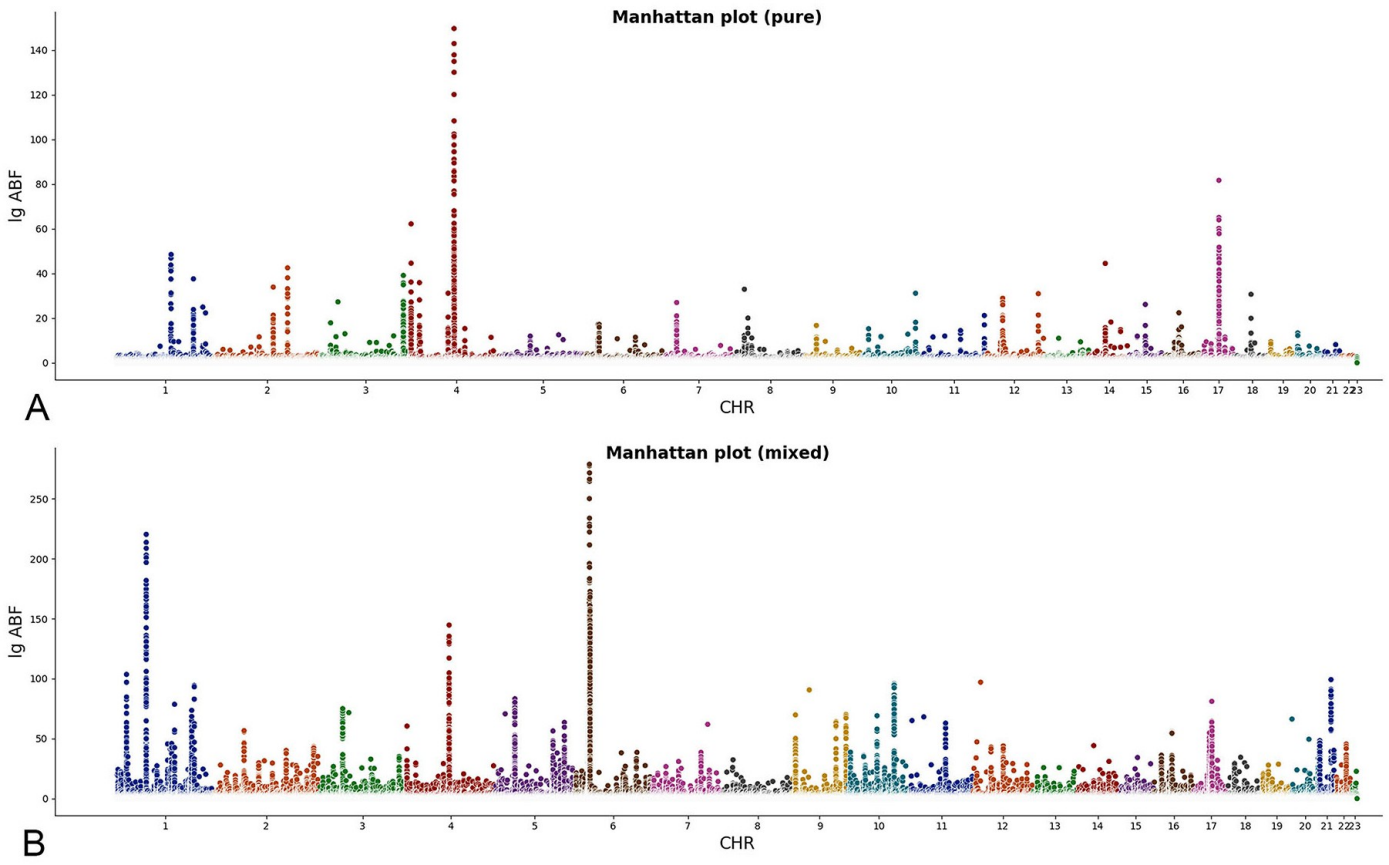
Manhattan plots. A. Results of pure meta-analysis, which includes 29 studies on PD. B. Results of mixed meta-analysis, which includes 59 studies on PD, inflammatory bowel disease (including its two subtypes: Crohn’s disease and ulcerative colitis), multiple sclerosis, and systemic sclerosis. lg ABF: log_10_ABF.

Through the websites accommodating GWAS datasets, including DistiLD [21] (http://distild.jensenlab.org), Open Targets Genetics [22] (https://genetics.opentargets.org/) and GWAS Catalog [23] (https://www.ebi.ac.uk/gwas/), 59 studies in which the summary statistics ($\hat{\beta }$ and $SE_{\hat{\beta }}$) are provided or can be calculated are included in this application. Tables 2 and 3 show detailed information about the studies included. A pure meta-analysis across 29 studies on PD is conducted firstly, and then all 59 studies are analyzed jointly to obtain a mixed association pattern. The effects of over 10 million variants are assessed through parallel computing. The Manhattan plots for both the pure pattern and the mixed pattern are shown in Fig 5.

**Table 2:** The information of studies on PD included in the application.

First author	Published year	Ancestry	Sample Size (cases/controls)
Maraganore DM [24]	2005	European (US)	332/332
Pankratz N [25]	2008	European (US)	857/867
Satake W [26]	2009	East Asian (Japan)	2,011/18,381
Simn-Snchez J [27]	2009	European	1,713/3,978
Sutherland GT [28]	2009	European (Australia)	331/296
Edwards TL [29]	2010	European (US)	1,752/1,745
Hamza TH [30]	2010	European (US)	2,000/1,986
Saad M [31]	2010	European	4,271/9,048
Do CB [32]	2011	European	3,426/29,624
Liu X [33]	2011	Ashkenazi Jewish	2,050/1,836
Nalls MA [34]	2011	European	5,333/12,019
Spencer C [35]	2011	European (UK)	1,705/5,175
Simn-Snchez J [36]	2011	European (Dutch)	772/2,024
Lill CM [37]	2012	World	16,452/48,810
Nalls MA [38]	2014	European (US)	13,708/95,282
Hill-Burns EM [39]	2014	European (US)	1,986/2,000
Foo JN [40]	2017	East Asian	5,125/17,604
Chang D [41]	2017	World	26,035/403,190
Bandres-Ciga S [42]	2019	European (Spain)	4,639/2,949
Blauwendraat C [43]	2019	World	17,996 cases
Nalls MA [44]	2019	European	33,674/449,056
Blauwendraat C [45]	2020	European	1,588/7,584
Alfradique-Dunham I [46]	2021	European	1,570/1,259
Backman JD [47]	2021	European (UK)	828/330,926
Jiang L [48]	2021	European (UK)	294/456,054
Rodrigo LM [49]	2021	European	5,167/5,366
Smeland OB [50]	2021	European	20,184/975,838
Sakaue S [51]	2021	European & East Asian (Japan)	2,978/653,168
CIDR dataset^1^	-	World	1,048/943

^1^ Details of the dataset can be found at https://www.ncbi.nlm.nih.gov/projects/gap/cgi-bin/study.cgi?study_id=phs000126.v2.p1

**Table 3:** The information of studies on other autoimmune disorders included in the application.

First author	Published year	Ancestry	Sample Size (cases/controls)
**inflammatory bowel disease**			
Anderson CA [52]	2011	European	6,687/19,718
Jostins L [53]	2012	European	12,924/21,442
Juli A [54]	2014	European	7,483/21,211
Liu JZ [55]	2015	European	25,273/26,715
Liu JZ [55]	2015	Iranian	548/342
Liu JZ [55]	2015	Indian	1,423/990
Liu JZ [55]	2015	East Asian	2,824/3,719
Ostrowski J [56]	2016	European (Poland)	1,118/582
Yang SK [57]	2016	East Asian (Korea)	1,505/4,041
de Lange KM [58]	2017	European	25,042/34,915
Backman JD [47]	2021	European (UK)	5,650/298,738
Dnerta HM [59]	2021	UK	4,101/480,497
Glanville KP [60]	2021	European (UK)	5,105/324,074
Jiang L [48] (Crohn’s disease)	2021	European (UK)	1,342/455,006
Jiang L [48] (ulcerative colitis)	2021	European (UK)	2,569/453,779
Sakaue S [51]	2021	European & East Asian (Japan)	5,685/590,936
Wu Y [61]	2021	European	7,045/449,282
**multiple sclerosis**			
Hafler DA [62]	2007	European	931/2,431
De Jager PL [63]	2009	European	2,624/7,220
Patsopoulos NA [64]	2011	European	5,545/12,153
Sawcer S [65]	2011	European	9,772/16,849
Beecham AH [66]	2013	European	14,498/24,091
Andlauer TF [67]	2016	European (German)	4,888/10,395
IMSGC [68]	2019	World	14,802/26,703
Backman JD [47]	2021	European (UK)	1,596/330,158
Glanville KP [60]	2021	European (UK)	1,683/324,074
Jiang L [48]	2021	European (UK)	775/455,573
**systemic sclerosis**			
Mayes MD [69]	2014	European	1,833/3,466
Lpez-Isac E [70]	2019	European	9,095/17,584
Jiang L [48]	2021	European (UK)	104/456,244

IMSGC: International Multiple Sclerosis Genetics Consortium

We can find PD is highly associated with several loci located within gene *SCNA* on chromosomes 4. A peak also appears on chromosome 17, around gene *MAPT*, *KANSL1*, and *NSF*. When other autoimmune disorders are included in the meta-analysis, the peaks appear on chromosomes 1 and 6 in the mixed pattern. Some significant variants can also be found on chromosome 4. Table 4 shows several SNPs identified in the analysis. Detailed results are available at https://figshare.com/articles/dataset/Table_S_zip/19179179.

**Table 4:** Some key variants identified in analysis.

	CHR	Gene	SNP
pure pattern	4	*SCNA*	rs2736990, rs356165, rs356203, rs356168, rs356200, rs2737029
	17	*MAPT*	rs17649553, rs62056850, rs8070723, rs62062279
	17	*KANSL1*	rs2532276, rs2696664, rs56406462, rs2532275, rs2532278, rs2532281, rs2696658
	17	*NSF*	rs199447, rs199451, rs169201
	1	*ASH1L*	rs71628662, rs145330152, rs12734374, rs145331499
	6	*BTNL2*	rs3763309, rs3763312, rs3793127, rs9268491, rs3817963
mixed pattern	6	*TSBP1-AS1*	rs3130320, rs926070
	6	*TSBP1*	rs2395150, rs6904636, rs3129908, rs502626, rs477005
	6	*BTNL2*	rs3129954, rs3129955, rs2076530, rs3817963
	6	*BAG6*	rs3130050, rs2242656, rs3130617, rs1077394
	1	*IL23R*	rs11465804, rs80174646, rs75328060, rs11805303, rs7539625, rs1004819
	4	*SCNA*	rs2736990, rs356165, rs356203, rs356168, rs356200, rs2737029

The results supports the previous reports on several essential loci related to PD, such as *SCNA*, *MAPT* and *KANSL1* [17]. Additionally, some degree of underlying relationships between PD and other autoimmune disorders are revealed by comparing the mixed pattern to the pure pattern, for they have some shared risk variants. For example, variants on *BTNL2* have high ABF in both the pure pattern (∼10^17^) and the mixed pattern (∼10^127^), and the subsets indicates that *BTNL2* is associated with all four disorders. For the top 4 variants on *BTNL2* in the mixed pattern, on average 66.7% studies on PD, 65.7% studies on inflammatory bowel disease, 100% studies on multiple sclerosis, and 62.5% studies on systemic sclerosis are included in the final subsets to calculate ABF. However, although variants on *SCNA* also have a high ABF value in both pure pattern and mixed pattern, we found most of the studies in the final subsets to calculate ABF are from those studies on PD and the ABF values remain similar (∼10^135^) in both patterns. In other words, *SCNA* has weaker associations with other autoimmune disorders. The same is also true for those variants on *MAPT*. These two loci may relate more to the diseases in nervous system instead of autoimmune disorders. On the contrary, variants on *KANSL1*, reported as a factor in the immune system [71], show associations with both PD and other autoimmune disorders. In breif, he results of the meta-analysis indicate the presence of potential biological pathways and functional interactions between PD and autoimmune disorders. Tools like GESLM can use the shared variants to further identify causal variants [72].

### 4.2 Software

We implement all the algorithms as an R package named GWASmeta. Besides, to help researchers to use SMetABF to quickly find key SNPs, we develop a web tool based on R Shiny as well. The requirements of the file uploaded can be found in the website. Multiple variants can be analyzed at once. This tool is accessible at https://sunjianle-sjtu.shinyapps.io/analycode.

## 5 Discussion

Meta-analysis has been widely conducted on GWAS data to discover essential loci associated with some complex genetic diseases during recent years [73–75], satisfying the requirements of large sample size in GWAS. However, the traditional *p*-value method used in meta-analysis is facing increasing criticisms. For example, it is not proper to use a single threshold since *p*-value is dependent on the MAF and sample size [10]. Moreover, the sophisticated relationships between different studies are tricky to deal with in traditional methods. FEM relies on the assumption that all studies in the meta-analysis share a common true effect size. The true effect size is allowed to vary in different studies in REM, but the detail information is hard to be included in the model. And the test for heterogeneity to determine whether FEM or REM should be used is often regarded poor in power [11]. Instead, the structure among different studies can be easily integrated into the Bayesian model as a prior. The BF compares the relative size between *P*(*H*_0_|*Y*) and *P*(*H*_1_|*Y*), and therefore is a better alternative to the *p*-value. Based on the Bayesian framework, a useful statistical model named MetABF has been proposed, which could easily measure the associations between multiple phenotypes and variants at the same time using GWAS summary statistics but confronts challenges in computation.

In this article, we propose SMetABF, an improved tool to attain the optimal ABF in a large-scale meta-analysis efficiently. Through simulation, we confirm that SSS is superior to MCMC in terms of speed, accuracy, and stability. To a certain extent, our improvements effectively overcome the calculation problems due to the increase in the number of studies included. We performe an application to PD and other autoimmune disorders, illustrating the effectiveness of SMetABF. With more research conducted on various traits among a larger population and the increasing accumulations of GWAS summary statistics, the large-scale multi-phenotypic meta-analyses will be possible through SMetABF. Another possible application is to analyze the effect size across different variants in one study, where *σ*_*i*_ represents the prior variation of the *i*-th variant on the outcome, and *ρ*_*i*,*j*_ stands for the linkages between different variants. Furthermore, since many traits related to some complex diseases are correlated, it is necessary to consider the effect of multiple loci on the outcome across a large number of studies simultaneously [76]. In this case, the prior correlation matrix **Σ** will transform to a three-dimensional array, which will bring more challenges in computation.

The method still confronts many challenges. The choice of prior parameters is an example. Sensitive analysis reveals that different values of *σ* and *ρ* will affect the ABF values but seem not to change the relative effect size between different variants. Besides, the considerable size of human genome still brings challenges in computation.

The pooled statistics derived through meta-analysis can be further used for other post-GWAS analysis, for example, to identify causal genes through statistical fine-mapping [77] or to infer the causal relationships between traits by Mendelian randomization [78]. GWAS summary statistics from different studies can be conveniently integrated to a powerful pooled statistic by SMetABF. We believe the method will benefit to the integration of previous studies and help to reveal the genetic mechanisms of complex diseases.
